# Preservation of the celiac branch of the vagus nerve reduces the incidence of postoperative diarrhea in gastric cancer: a cohort study

**DOI:** 10.1186/s12957-024-03370-0

**Published:** 2024-04-06

**Authors:** Hao Guo, WeiFeng Sun, HaiTao Duan, Chi Zhang, MaoHua Wei, Pin Liang, Xiang Hu, Liang Cao

**Affiliations:** Department of General Surgery, The First Affiliated Hospital, Dalian Medical University, Dalian, Liaoning Province 116011 China

**Keywords:** Celiac branch preservation, Gastrectomy, Laparoscopy, Stomach neoplasms/surgery, *Vagus Nerve, Laparoscopy-assisted distal gastrectomy

## Abstract

**Background:**

To investigate the short-term and long-term outcomes of preserving the celiac branch of the vagus nerve during laparoscopic distal gastrectomy.

**Methods:**

A total of 149 patients with prospective diagnosis of gastric cancer who underwent laparoscopic-assisted distal gastrectomy (LADG) combined with Billroth-II anastomosis and D2 lymph node dissection between 2017 and 2018 were retrospectively analyzed. The patients were divided into the preserved LADG group (P-LADG, *n* = 56) and the resected LADG group (R-LADG, *n* = 93) according to whether the vagus nerve celiac branch was preserved. We selected 56 patients (P-LADG, *n* = 56) with preservation of the celiac branch of the vagus nerve and 56 patients (R-LADG, *n* = 56) with removal of the celiac branch of the vagus nerve by propensity-matched score method. Postoperative nutritional status, weight change, short-term and long-term postoperative complications, and gallstone formation were evaluated in both groups at 5 years of postoperative follow-up. The status of residual gastritis and bile reflux was assessed endoscopically at 12 months postoperatively.

**Results:**

The incidence of diarrhea at 5 years postoperatively was lower in the P-LADG group than in the R-LADG group (*p* < 0.05). In the multivariate logistic analysis, the removal of vagus nerve celiac branch was an independent risk factor for the occurrence of postoperative diarrhea (odds ratio = 3.389, 95% confidential interval = 1.143-10.049, *p* = 0.028). In the multivariate logistic analysis, the removal of vagus nerve celiac branch was an independent risk factor for the occurrence of postoperative diarrhea (odds ratio = 4.371, 95% confidential interval = 1.418-13.479, *p* = 0.010).

**Conclusions:**

Preservation of the celiac branch of the vagus nerve in LADG reduced the incidence of postoperative diarrhea postoperatively in gastric cancer.

**Trial registration:**

This study was registered with the Ethics Committee of the First Affiliated Hospital of Dalian Medical University in 2014 under the registration number: LCKY2014-04(X).

## Introduction

Gastric cancer, ranking fifth in new incidence and fourth in mortality worldwide in 2020, [1] continues to be a major cause of cancer-related mortality accounting for 8% in 2020. Morgan et al. [2] . Improvements in screening technology and gastroscopy in recent years have contributed to a gradually increasing detection rate of early gastric cancer, which leads to a significant reduction in mortality rate [3, 4]. As a result, the focus of gastric cancer surgery has begun to shift towards improving the postoperative quality of life (QOL) for patients.

Laparoscopic-assisted gastric cancer surgery has been validated for its various advantages over traditional open surgery in many ways [5, 6]. Compared to open distal gastrectomy (ODG), laparoscopic assisted distal gastrectomy (LADG) has the advantages of shorter operative time, reduced intraoperative bleeding, faster recovery of intestinal function and shorter postoperative hospital stay [7, 8]. Studies have also confirmed its safety in terms of long-term prognosis and tumor recurrence [9, 10]. However, as conventional distal gastrectomy involves the removal of a large portion of the gastric body and pylorus, division of the anterior and posterior vagus nerve trunks, and extensive lymph node dissection, postoperative patients often suffer from complications such as diarrhea, dumping syndrome, and gallstones. Therefore, it becomes an additional goal of surgery to maximize the preservation of organ function and improve the postoperative quality of life of patients while ensuring radicality.

Initially appeared for the treatment of gastric ulcer, vagus nerve preserving gastrectomy is now focusing on the reduction of the postoperative complications of gastric cancer. By preserving the function of the vagus nerve, this approach aimes to improve the quality of life of patients after surgery while mitigating complications. In previous studies, postoperative complications, such as postoperative diarrhea and loss of appetite, were found to be less frequent in patients with preserved vagus nerve gastrectomy than in those without preservation [11]. The advantages were seen including faster recovery of intestinal function [10] and maintenance of postoperative weight [12]. In addition, it has been demonstrated that preservation of the hepatic branch of the vagus nerve significantly reduces the incidence of postoperative gallstones [13, 14]. However, vagus nerve preserving gastrectomy, with all these benefits, is still considered as a relatively new technique which has not been generalized in clinic, for which further validation and confirmation are in need. In this study, we intended to investigate the efficacy of preserving the celiac branch in distal gastrectomy combined with B-II anastomosis by conducting the postoperative follow-up assessments for patients who underwent distal gastrectomy with preserved vagus nerve celiac branch.

## Materials and methods

### Patient characteristics

The study conducted a prospective analysis of 149 patients diagnosed with gastric cancer preoperatively by CT, gastroscopy and ultrasound between 2017 and 2018. Laparoscopic distal gastrectomy combined with Billroth-II type anastomosis was performed at the Department of Gastroenterology, The First Hospital of Dalian Medical University. Intraoperative D2 lymph node dissection was performed adhered to the Japanese guidelines for the treatment of gastric cancer [15]. After obtaining informed consent from the patients, a computerized random sampling method was used to decide which patients would have the celiac branch of the vagus nerve preserved. To reduce the impact of potential confounders, we employed propensity score matching. This statistical technique aimed to balance the covariates between the treatment groups, ensuring that any observed differences in the outcome were more likely to be attributed to the treatments themselves rather than the influence of confounding factors. Demographic and clinical characteristics of the patients, such as age, BMI, size, Weight, gender, and diabetes, were collected for both groups. We performed 1:1 greedy nearest neighbor matching with a caliper of 0.2. The method functionally relied on the R package MatchIt. A distance was computed between unit of one group and another, and, one by one, each unit was assigned a control unit as a match. The patients were divided into preserved LADG group (P-LADG, *n* = 56) and resected LADG group (R-LADG, *n* = 93). Patient inclusion criteria were as follows: (1) preoperative diagnosis of gastric cancer; (2) age below 80 years (as gastrointestinal function would reduce in elderly patients over 80); (3) absence of other malignant tumors; (4) no history of radiotherapy and chemotherapy before and after surgery; (5) no presence of gallstones and no cholecystectomy before surgery; (6) absence of serious diseases in cardiovascular, respiratory, liver and kidney systems, or psychiatric abnormalities. Ultimately, 112 patients were divided into preserved LADG group (P-LADG, *n* = 56) and resected LADG group (R-LADG, *n* = 56). Patient inclusion criteria were as follows: (1) preoperative diagnosis of gastric cancer; (2) age below 80 years (as gastrointestinal function would reduce in elderly patients over 80); (3) absence of other malignant tumors; (4) no history of radiotherapy and chemotherapy before surgery; (5) no presence of gallstones and no cholecystectomy before surgery; (6) absence of serious diseases in cardiovascular, respiratory, liver and kidney systems, or psychiatric abnormalities. All patients with stage T3 and above underwent postoperative chemotherapy. Patients and their families voluntarily participated in this study have signed the informed consent forms. The study was reviewed and approved by the ethics committee of the hospital.

### Surgical procedures

**Fig. 1:**
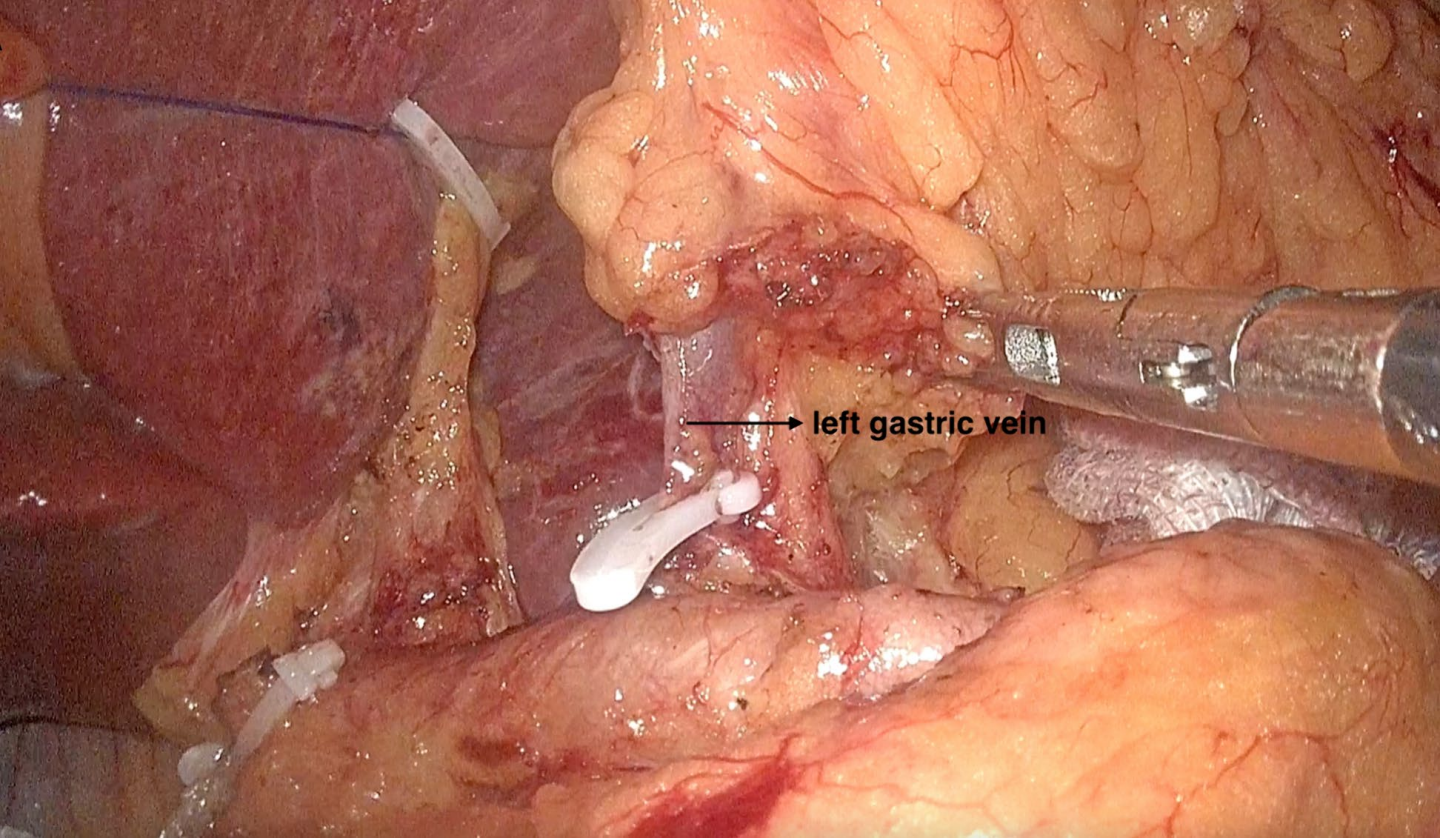
Disconnect the left gastric vein

**Fig. 2:**
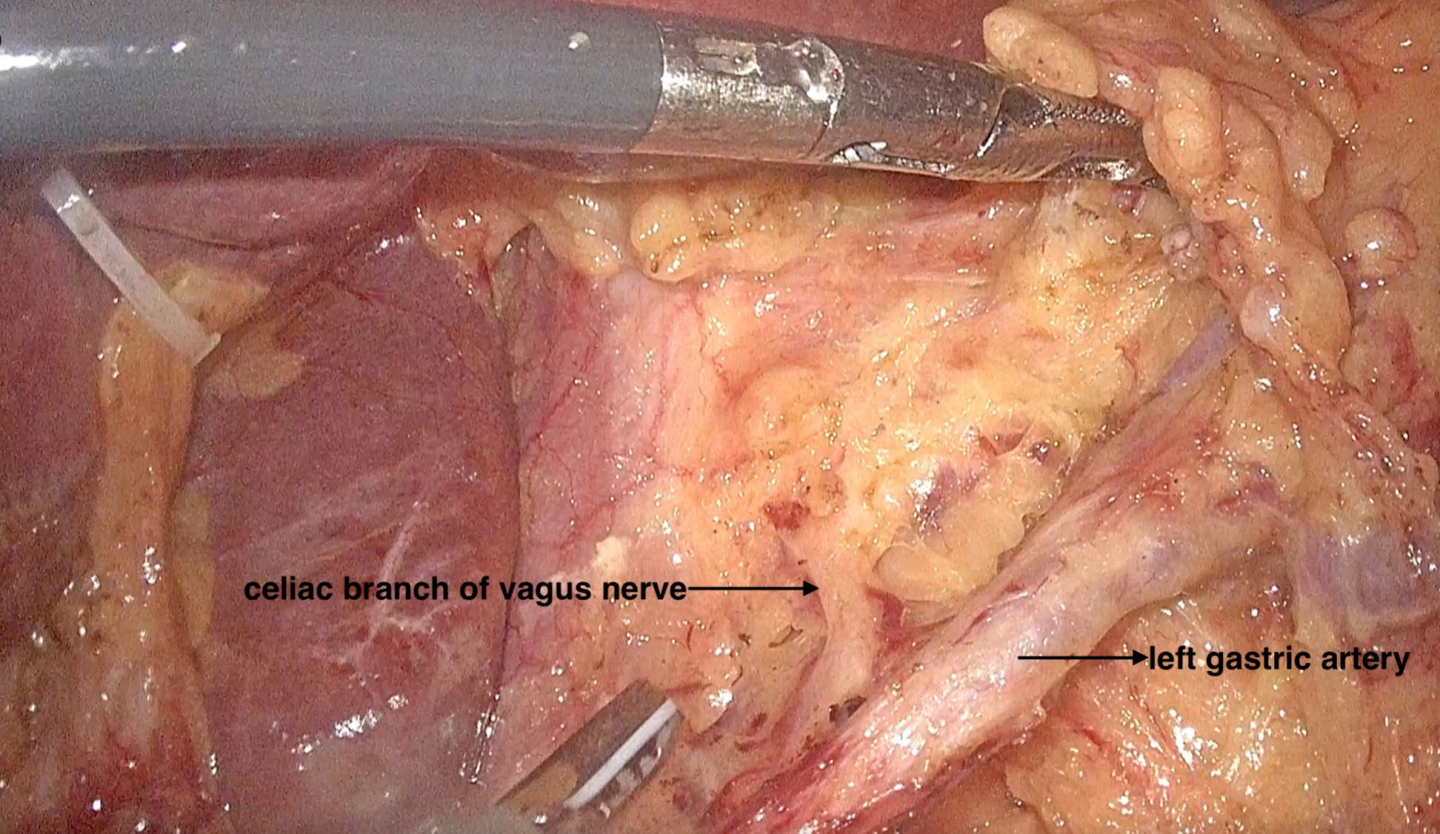
With the exposure of the right lateral fold of the diaphragm and the anterolateral aspect of the abdominal esophagus, the celiac branch of the vagus nerve extending along the posterior wall of the abdominal esophagus was identified

**Fig. 3:**
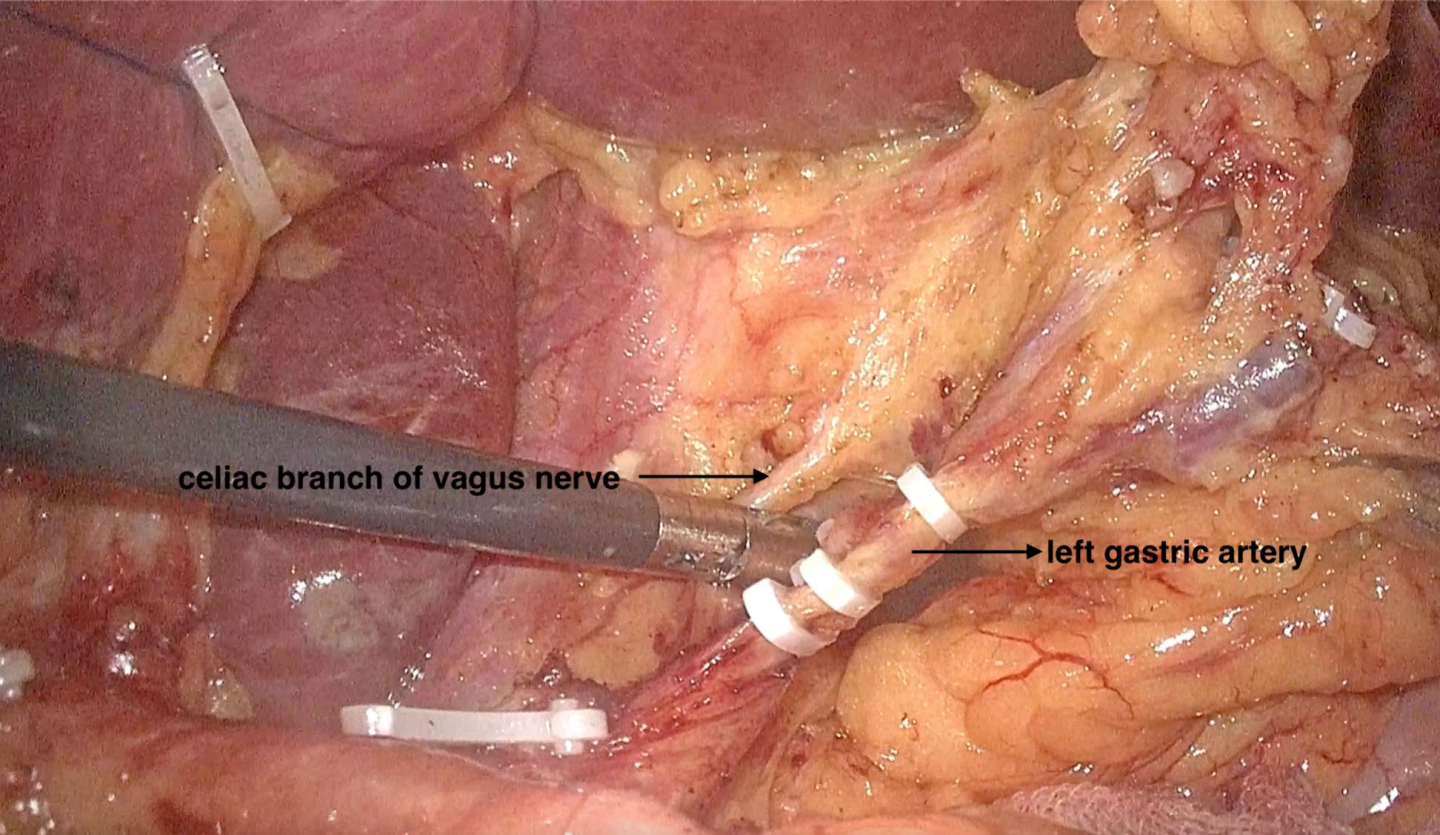
The celiac branch of the vagus nerve was isolated at the root of the left gastric artery, and the left gastric artery was dissected here

The surgery was performed with the patient in a supine split-legged position with hands placed on both sides. Five poke cards were inserted in the standard laparoscopic gastric surgery position. A pneumoperitoneum was established using CO_2_ gas, while maintaining the abdominal pressure at 14 mmHg. After laparoscopic exploration of the abdominal cavity, the left lobe of the liver was elevated. A small hole of approximately 2.0 cm was incised through the lesser omentum close to the liver using an ultrasonic scalpel, which was then secured using a 1–0 ethilon suture and small hem-o-lok clips. In this way, the liver was suspended. The greater omentum was preserved, and the gastrocolic ligament was incised. The greater curvature of the stomach was freed 3.0 cm beyond the lateral vascular arch of the greater curvature. The right vessels of the gastric omentum were released circumferentially. Clearance of group 6 lymph nodes was performed, and the gastrocolic ligament was dissociated extending to the lower pole of the spleen and its root. After clamping the left vessel of the gastric omentum and the first branch of the short gastric vessels, the 4sa and 4sb lymph nodes were cleared. Above the pylorus, the right gastric artery was isolated and ligated, and group 5 lymph nodes were contoured. At a distance of 3.0 cm from the pylorus, the duodenum was dissected intralumbrally. The duodenum was divided near the pylorus using anendoscopic liner stapler (Ethicon, USA). The clearance of lymph nodes of groups 8a and 12a were performed along the common hepatic artery and intrinsic hepatic artery, followed by the clearance of lymph nodes of group 9 as well as those of group 11p along the splenic artery. The left gastric vein and the left gastric artery were visualized circumferentially and the lymph nodes of group 7 were contoured, and then the left gastric vein was dissected (Fig. 1). Next, the right diaphragmatic pedicle and the anterolateral aspect of the abdominal esophagus were isolated, and the celiac branch of the vagus nerve, which runs along the posterior wall of the esophagus, was identified (Fig. 2). The celiac branch of the vagus nerve was isolated at the root of the left gastric artery. When separating the celiac branch of the vagus nerve from the left gastric artery, attention needs to be paid to the variation of the posterior trunk of the vagus nerve from the celiac branch. The celiac branch of the vagus nerve is then drawn to the right, and the posterior gastric branch is then separated from the celiac branch. The left gastric artery was dissected at the root of the left gastric artery and the celiac branch of the vagus nerve was preserved (Fig. 3). The anterior and posterior vessels were dissected branch by branch along the subcardial, and the lymph nodes of groups 1 and 3 were cleared. The distal stomach was dissected 5 cm from the lateral end of the tumor orifice, and Billroth-II + Braun was performed under full laparoscopy. Digestive tract reconstruction was performed as the last step.

### Research indicators

1. Patient characteristics
  Patients in both groups were followed up for 5 years. General information of patients was collected, which included age, gender, BMI, weight, tumor size, and whether they had diabetes.
2. Perioperative related indicators and pathologic data
  The operation period, bleeding volume, time to first postoperative flatus, time to first feeding, time to drainage tube removal, duration of hospital stay, number of lymph nodes dissected, number of positive lymph nodes, presence of vascular or neurologic invasion, degree of tumor differentiation, and occurrence of short-term postoperative complications were recorded for both groups.
3. Endoscopy, long-term complications and indicators of nutritional status
  Postoperative examinations were performed according to the Japanese guidelines for the treatment of gastric cancer [15]. Grades of residual gastritis, residual food and bile reflux were determined based on the classification of "residue, gastritis, bile", [16] and grades 2 or higher were recorded. The presence of gallbladder stone formation was assessed through observation by abdominal ultrasound and abdominal CT. Patients underwent evaluations for the presence of dumping symptoms and diarrhea at 5 years after surgery. Diarrhea was defined as a postoperative change in bowel habits with 2 or more loose or watery bowel movements within one week [17]. Dumping syndrome was defined as the presence of any of the following symptoms after a meal: palpitation, pallor, abdominal pain, tenderness, sweating, or dizziness [18]. Postoperative nutritional status was assessed by preoperative and 1-year postoperative measurements of serum albumin and total protein as well as weight changes.

### Statistical analysis

SPSS 25.0 software was used for statistical analysis. We used t-test (for measurement data conforming to normal distribution) or rank sum test (Mann–Whitney U) test (for data not conforming to normal distribution) for comparison of characteristics of patients between groups, while Chi-square test was used for comparison of count data. The prognostic factors were analyzed mainly by multivariate logistic regression model. Some of the data and images were analyzed and plotted through the R language. A significance level of p value < 0.05 was considered statistically significant.

## Result

At the timepoint of 5 years postoperative follow-up, the incidence of diarrhea at 5 years postoperatively was lower in the P-LADG group than in the R-LADG group (*p* < 0.05). In the multivariate logistic analysis, the removal of vagus nerve celiac branch was an independent risk factor for the occurrence of postoperative diarrhea (odds ratio = 3.389, 95% confidential interval = 1.143–10.049, *p* = 0.028). In the multivariate logistic analysis, the removal of vagus nerve celiac branch was an independent risk factor for the occurrence of postoperative diarrhea (odds ratio = 4.371, 95% confidential interval = 1.418–13.479, *p* = 0.010).

Data for 56 patients in the P-LADG group and 56 patients in the R-LADG group were selected using the propensity score-matching method. The characteristics of the patients are shown in Table 1. No difference was observed between the two groups in terms of age, gender, tumor size, BMI, weight, and comorbidities.

**Table 1:** Baseline information and characters of patients

Variable	R-LADG(*n* = 56)	P-LADG(*n* = 56)	*P*
Age(y)	**63(56.0,67.0)**	**61.5(57.3,66.0)**	**0.802**
Gender,*n*(%)			
Male	**40(71.4)**	**40(71.4)**	**1**
Female	**16 (28.6)**	**16(28.6)**	
Size(cm)	**3.0(2.2, 4.5)**	**3.0(2.4, 4.0)**	**0.691**
BMI(kg/m^2^)	**24.3 ± 3.7**	**23.9 ± 3.8**	**0.609**
Diabetes(%)			**0.740**
Y	**4(7.1)**	**6(10.7)**	
N	**52(92.9)**	**50(89.3)**	
Weight(kg)	**68.9 ± 12.7**	**68.2 ± 12.1**	**0.747**

The results of the comparison of surgery-related information and pathological data between the two groups of patients are shown in Table 2. There were no significant differences between the two groups in terms of operative time, intraoperative blood loss, time to first anal ventilation or first feeding, postoperative hospitalization duration, number of lymph nodes dissected, number of positive lymph nodes and occurrence of short-term complications. Although the time to drainage removal was greater in the P-LADG group than in the R-LADG group, there was no statistical significance (9.0 versus 8.0, *p* = 0.053). There was no significant difference in the incidence of short-term postoperative complications including anastomotic leakage and wound infection between the two groups (*p* > 0.05). Similarly, in the comparison of pathological data between the two groups of patients, there were no significant differences in their depth of tumor infiltration, degree of tumor differentiation, tumor lympho-vascular invasion and nerve invasion.

**Table 2:** Surgical information, pathological data and short-term complications in the R- LADG group and the P-LADG group

Variable	R-LADG(*n* = 56)	P-LADG(*n* = 56)	*p*
Operation time(min)	260.0 (221.3, 295.0)	270.0 (225.8, 295.0)	0.753
Blood loss(ml)	20.0 (10.0, 30.0)	20.0 (10.0, 20.0)	0.985
Time to flatus(day)	4.0 (3.0, 5.0)	3.0 (3.0, 4.0)	0.993
Time to intake(day)	5.0 (4.0, 7.0)	5.0 (4.0, 7.0)	0.290
Time to removal drainage(day)	8.0 (7.0, 9.8)	9.0 (7.0, 11.0)	0.148
Hospitalization time(day)	9.0 (8.0, 11.0)	10.0 (8.0, 12.0)	0.454
Anastomotic Leakage(%)	1(1.1)	0(0)	1.00
Wound infection(%)			0.528
Yes	2 (3.6)	0 (0)	0.495
No	54 (96.4)	56 (100)	
Retrieved LN(number)	28.0 (16.0, 36.8)	25.0 (17.0, 32.8)	0.430
Positive LN (number)	0 (0.0, 1.0)	0 (0.0, 2.0)	0.225
Lympho-vascular invasion(%)			
Yes	12 (21.4)	13 (23.2)	0.820
No	44 (78.6)	43 (76.8)	
Neural-invasion(%)			
Yes	12 (21.4)	18 (32.1)	0.286
No	44 (78.6)	38 (67.9)	
Degree of tumor differentiation(%)			0.158
Poorly differentiated	28 (50)	18 (32.1)	
Moderately differentiated	23 (41.1)	31 (55.4)	
Well differentiated	5 (8.9)	7 (12.5)	
pT stage(%)			0.287
T1a	21 (37.5)	13 (23.2)	
T1b	9 (16.1)	15 (26.8)	
T2	12 (21.4)	9 (16.1)	
T3	9 (16.1)	10 (17.9)	
T4	5 (8.9)	9 (16.1)	

**Fig. 4:**
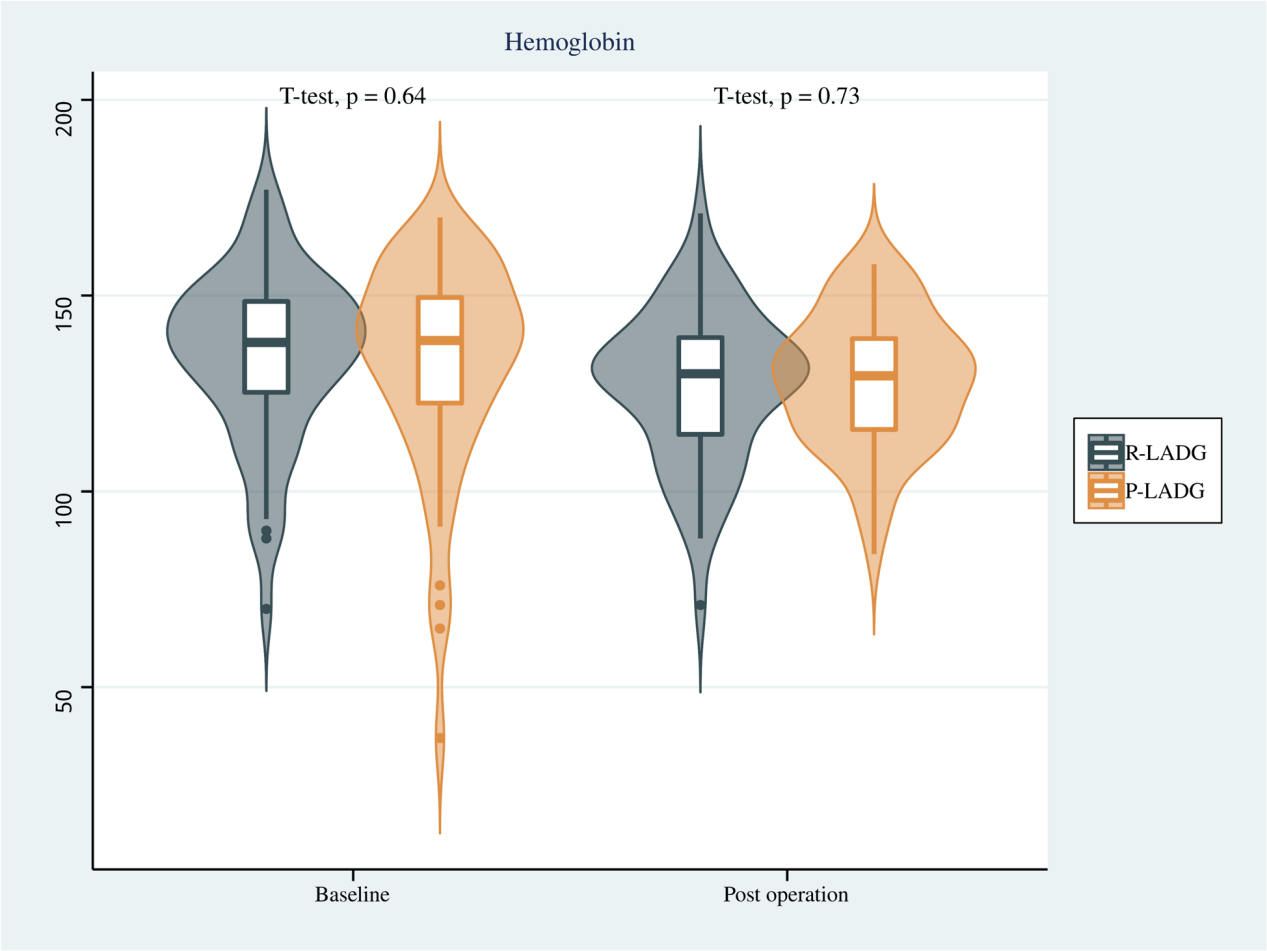
Comparison of hemoglobin of patients between the R-LADG group and the P-LADG group at baseline and at 1 year post surgery

**Fig. 5:**
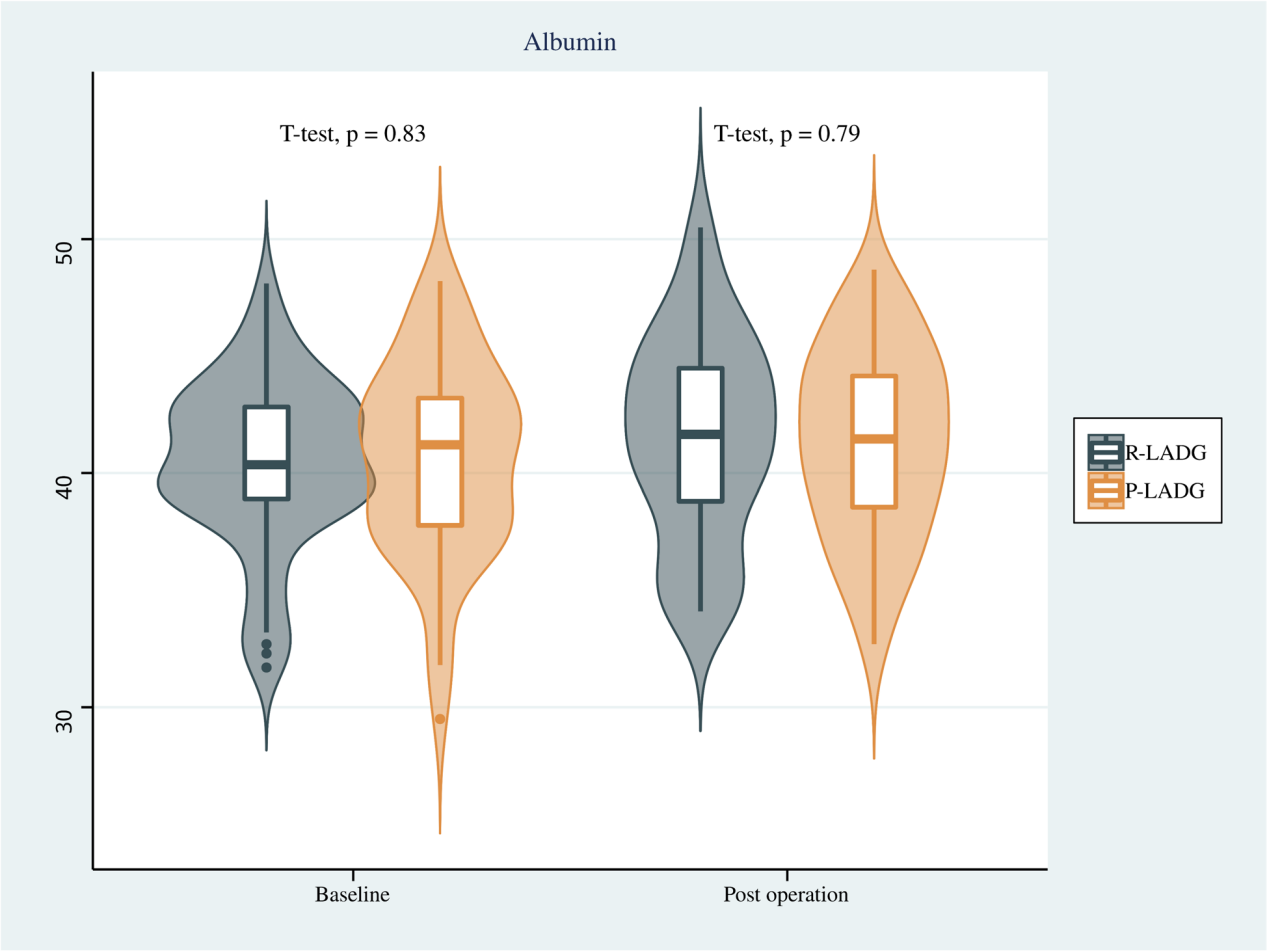
Comparison of albumin of patients between the R-LADG group and the P-LADG group at baseline and at 1 year post surgery

**Fig. 6:**
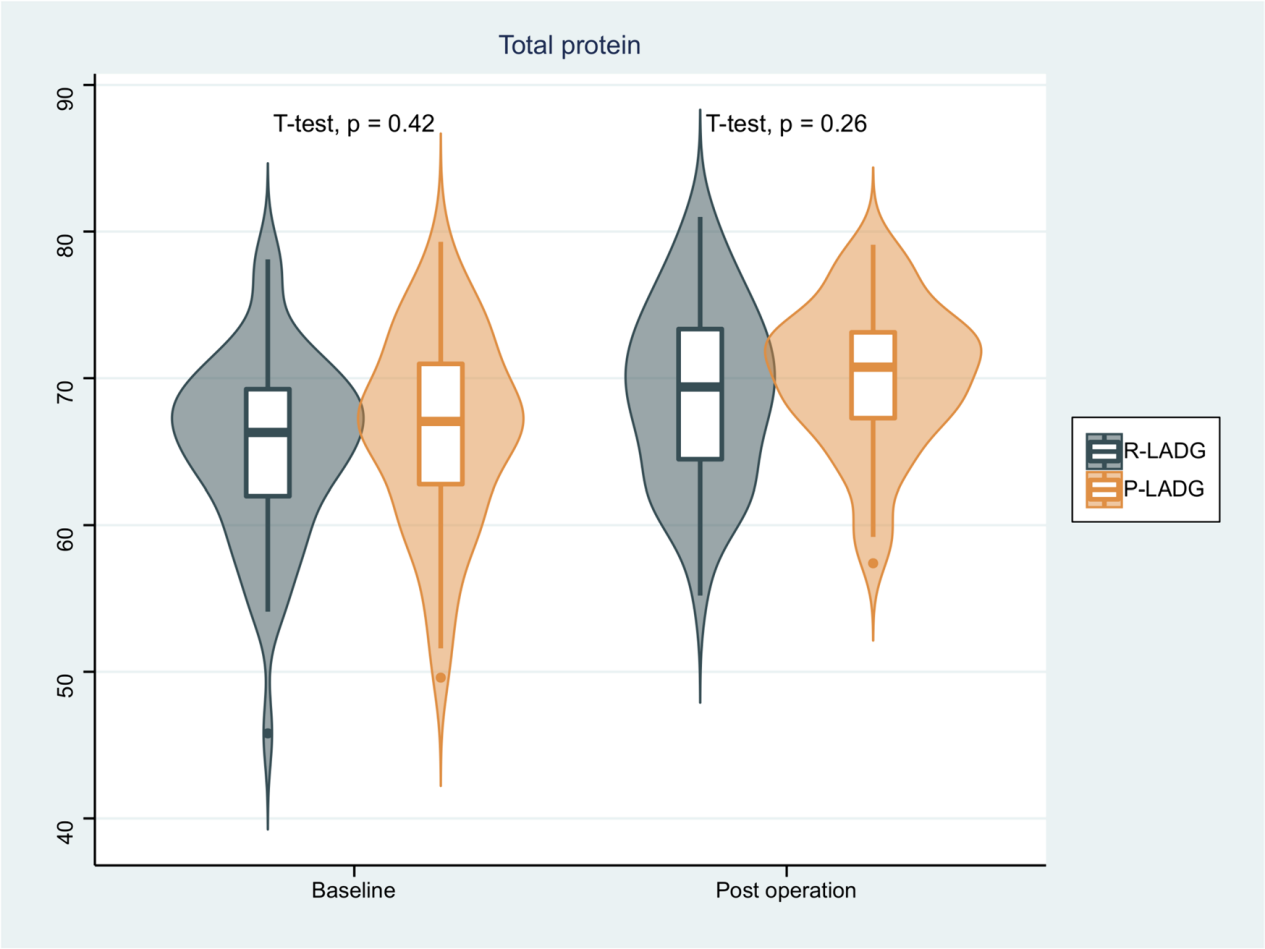
Comparison of total protein of patients between the R-LADG group and the P-LADG group at baseline and at 1 year post surgery

**Fig. 7:**
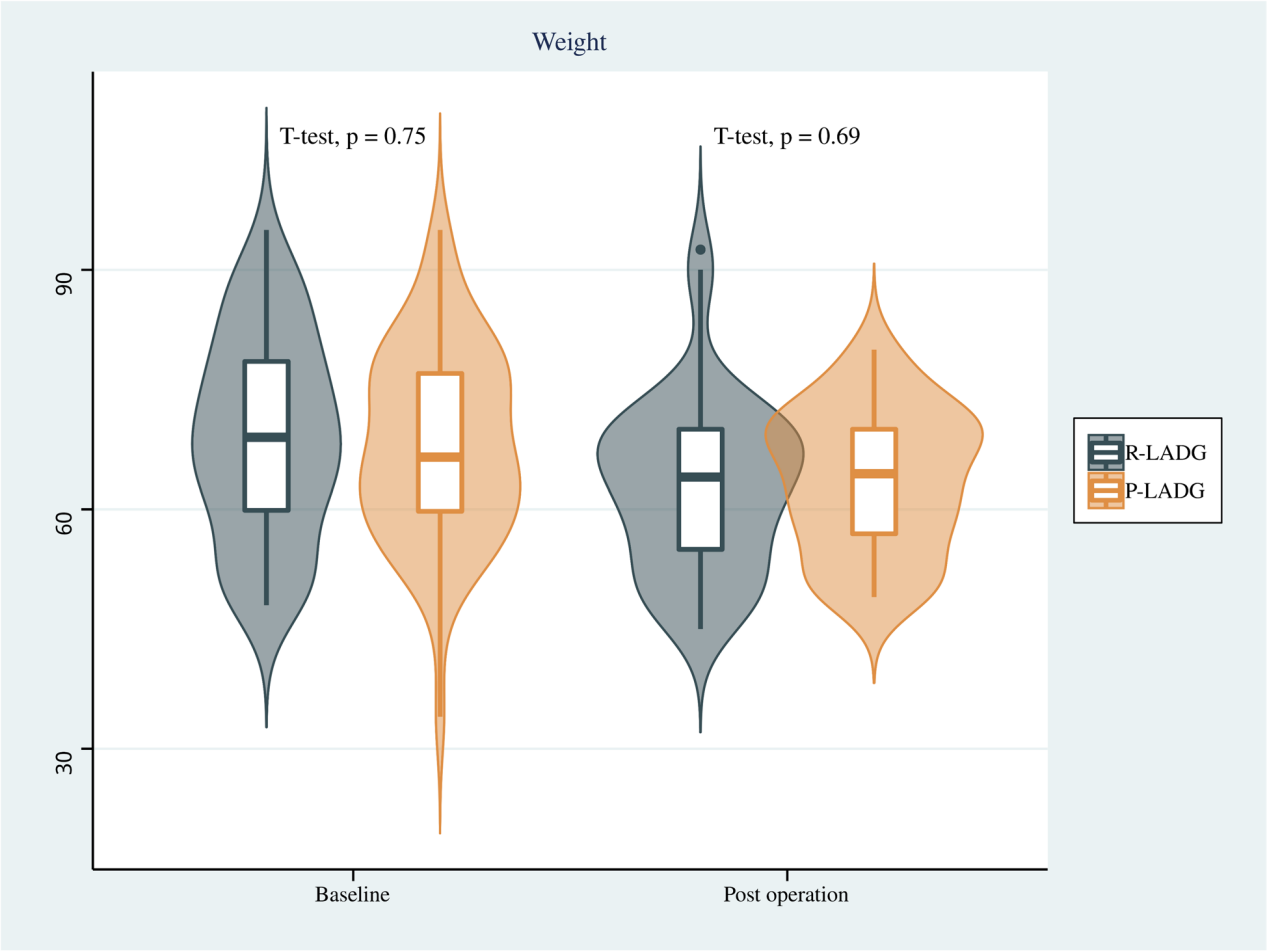
Comparison of body weight of patients between the R-LADG group and the P-LADG group at baseline and at 1 year post surgery

Endoscopy findings showed that there was no significant difference in the incidence of residual gastritis between the two groups, and similarly, there was no significant difference in residual food or bile reflux between the two groups. Regarding indicators of nutritional status, no significant differences were found in the changes of hemoglobin, albumin, total protein and body weight between the two groups before the surgery and one year after surgery (*p* > 0.05). Results are shown in Table 3 and Figs. 4, 5, 6 and 7.

**Table 3:** Comparison of endoscopic findings 1 year after surgery for patients in the R- LADG group and the P-LADG group

Variable	R-LADG(*n* = 56)	P-LADG(*n* = 56)	*p*
Remnant gastritis(%)			0.122
Yes	46 (82.1)	39 (69.6)	
No	10 (17.9)	17 (30.4)	
Residual food(%)			0.112
Yes	9 (16.1)	16 (28.6)	
No	47 (83.9)	40 (71.4)	
Bile reflux(%)			0.405
Yes	9 (16.1)	6 (10.7)	
No	47 (83.9)	50 (89.3)

Regarding the long-term complications, diarrhea occurred in only 5 patients in the P-LADG group while 24 cases were observed in the R-LADG group (8.9% vs. 25.8%, *p* = 0.012). Regarding the long-term complications, diarrhea occurred in only 5 patients in the P-LADG group while 17 cases were observed in the R-LADG group (8.9% vs. 30.4%, *p* = 0.009). We used the Common Terminology Criteria for Adverse Events (CTCAE—Version 5.0) to evaluate the severity of diarrhea [19]. No patient in either group had diarrhea of grade 3 or higher, and only 1 patient in the P-LADG group had grade 2 diarrhea, while 5 patients in the R-LADG group had grade 2 diarrhea. There were no significant differences in occurrence of postoperative dumping syndrome or gallstone formation between the two groups (*p* > 0.05), as shown in Table 4.

**Table 4:** Comparison of occurrence of long-term complications in patients between the R-LADG group and the P-LADG group

Variable	R-LADG(*n* = 56)	P-LADG(*n* = 56)	*p*
Dumping syndrome(%)			0.611
Yes	3 (5.4)	1 (1.8)	
No	53 (94.6)	55 (98.2)	
Diarrhea(%)			0.009
No	39 (69.6)	51 (91.1)	
Grade 1	12 (21.4)	4 (7.1)	
Grade 2	5 (8.9)	1 (1.8)	
Gallstones(%)			0.391
Yes	9 (16.1)	5 (8.9)	
No	47 (83.9)	51 (91.1)	

Binary logistic regression equations were constructed by selecting indicators with P values less than 0.2. The results revealed that removal of the vagus nerve celiac branch was an independent risk factor for the development of postoperative diarrhea (odds ratio = 3.389, 95% confidential interval = 1.143–10.049, *p* = 0.028), as shown in Table 5. Binary logistic regression equations were constructed by selecting indicators with P values less than 0.2. The results revealed that removal of the vagus nerve celiac branch was an independent risk factor for the development of postoperative diarrhea (odds ratio = 4.371, 95% confidential interval = 1.418–13.479, *p* = 0.010)., as shown in Table 5.

**Table 5:** Multivariate analysis of the morbidity of postoperative diarrhea

**Variable**		**B**	**SE**	**Wald**	***p***	**OR**	**95%CI**
**Group**	P-LADG*						
	R-LADG	1.475	0.575	6.592	0.010	4.371	1.418 -13.479
**Time to removal drainage**		-0.087	0.100	0.759	0.384	0.917	0.754–1.115
**Degree of differentiation**		0.033	0.399	0.007	0.935	1.003	0.473–2.257
**Remnant gastritis**		-0.218	0.642	0.115	0.734	0.804	0.228–2.830
**Residual food**		0.404	0.618	0.428	0.513	1.498	0.446–5.029

*Group being compared

## Discussion

As the diagnosis efficacy and survival rate of early gastric cancer have been improved, the focus of surgery has gradually shifted from ensuring curative radicality to embracing minimally invasive, individualized, and precise techniques. The major goal of surgical procedures nowadays is to limit the extent of resection, preserve nerve function, maintain the anatomy of the stomach and improve postoperative quality of life while guaranteeing a radical resection of the lesion.

The vagus nerve is the most widely distributed and longest traveling mixed type of nerve in the body, which includes afferent and efferent nerves known as sensory neurons and motor neurons, respectively. Approximately 90% of the inferior phrenic vagus nerve consists of afferent neurons. The anterior trunk of the vagus nerve runs ventrally down the esophagus which divides into the anterior gastric branch and the hepatic branch at the cardia, while the posterior trunk is located in the dorsal segment of the esophagus which divides into the posterior gastric branch and the ventral branch at the cardia. The vagus nerve regulates the interdigestive function of gallbladder, the sphincter of Oddi, and the gastroduodenal motor pattern, where the celiac branch innervates the digestive tract spanning from the duodenum to the transverse colon and transmits information to the hypothalamus. Miao et al. [20]. It has been found that the digestive motorcycle of the gastroduodenal, gallbladder and sphincter of Oddi muscles increased significantly after the vagus nerve celiac branch was removed [21]. Currently, laparoscopic distal gastrectomy remains the primary surgical procedure for distal gastric cancer. However, the complex anatomy of the vagus nerve, which is often intertwined with blood vessels and surrounded by lymph nodes, raises the difficulty of the operation. As a result, the vagus nerve is often removed while the blood vessels are treated, which in turn leads to postoperative complications such as digestive disorders, gallbladder stones and diarrhea.

The complete dissection of lymph nodes during vagus nerve preserving gastrectomy is regared as a challenging task. In the study by Kong et al., [22] the metastasis rate of NO.5 and NO.6 lymph nodes was only 0.46% and 0.9% for early gastric cancer with tumors distal to the incisional margin exceeding 6 cm. However, the NO.5 and NO.6 lymph nodes were often left uncontoured or incompletely contoured in order not to damage the pyloric branch. The celiac branch of the vagus nerve runs in the gastro-pancreatic fold along the left gastric artery to its root, which is surrounded by the NO.7 lymph node. In addition, since the peripheral lymph nodes are located outside the vascular nerve tegument, it is theoretically possible to identify the level of the lymph nodes and the nerve tegument when preserving the nerve, and to completely contour the lymph nodes for protecting the nerve especially with the aid of the magnifying effect of laparoscopy.

With the increasing maturity of laparoscopic techniques, preservation of the autonomic nerve during laparoscopic gastrectomy has shown no substantial influence on short-term surgical outcomes and long-term patient survival. Several studies comparing surgical outcomes such as operative time, intraoperative blood loss, number of cleared lymph nodes, short-term postoperative complications, and duration of hospital stay between surgery with and without preservation of the vagus nerve have shown no significant differences [13, 17, 23, 24]. In terms of long-term survival, the 5-year recurrence-free survival rates were 99.1% and 97.1% (*p* > 0.05) for patients with and without preserved abdominal branches, respectively, in the study by Furukawa et al. [23]. In the study by Seong-Ho Kong et al., [13] the 3-year recurrence-free survival rates were 100.0% and 97.8% in the preserved distal vagus nerve gastrectomy group versus non-preserved group (*p* > 0.05). Liu et al. [10]. Similarly, in this study, the 5-year survival rates of the vagus nerve celiac branch preserved and unpreserved groups were 96.4% and 94.6%, respectively (*p* > 0.05), with no statistically significant difference. It is evident that vagus nerve preserving gastrectomy is safe and feasible under the proficient application of laparoscopic technique.

In terms of long-term complications, kazuyuki et al. [24] found that the incidence of postoperative gallstones was significantly lower in the vagus nerve-preserved group than in the unpreserved group (*p* < 0.05). Yamada et al. [17] reported that dumping syndrome occurred in only 2 (2.9%) patients after preserved distal celiac branch gastrectomy compared to 14 patients (15.7%) in the unpreserved group (*p* < 0.05). The incidence of postoperative diarrhea, bile reflux, and gastroparesis was significantly lower in patients in the celiac branch preserved group than in the unpreserved group in a previous report by the authors' research team(*p* < 0.05) [10]. Similarly, in a randomized controlled trial by Kim et al., [11] the incidence of diarrhea at 3 months versus 12 months postoperatively was significantly lower in patients in the vagus nerve preserved group than in the unpreserved group (*p* < 0.05). A study by Ichiro Uyama et al., [25] also showed that preservation of the celiac branch of the vagus nerve could reduce the incidence of postoperative diarrhea. In addition, T Ichikura et al., [26] suggested that injury to the peri-abdominal arterial plexus during lymph node dissection of the peri-abdominal artery may lead to postoperative diarrhea. In this study, the incidence of diarrhea was also lower in patients in the resected group than in the unreserved group (8.9% versus 25.8%, *p* < 0.05). In this study, the incidence of diarrhea was also lower in patients in the resected group than in the unreserved group (8.9% versus 30.4%, *p* < 0.05). However, in our study, the incidence of dumping syndrome and gallstones was not found to be significantly different in the two groups of patients (*p* > 0.05). Dumping syndrome is mainly caused by the release of bradykinin and serotonin, which triggers a significant fluid shift from blood vessels to the intestinal lumen, causing a reduction in circulating blood volume [17]. As the hepatic branch of the vagus nerve controls the contraction and secretion of the gallbladder, preserving the hepatic branch of the vagus nerve facilitates bile excretion and prevents bile stagnation, thus reducing the formation of gallstones. The celiac branch of the vagus nerve is widely distributed in the stomach, duodenum, jejunum, pancreas and other organs of the upper gastrointestinal tract, regulating gastrointestinal peristalsis and secretion of intestinal fluids and other body activities. The vagus nerve may also regulate gastrointestinal function by influencing the secretion of gastrointestinal hormones. Previous studies have reported a higher incidence rate of postoperative diarrhea in patients who undergo gastrectomy with vagal trunk dissection [27] because of the rapid transport and malabsorption of nutrients from the intestine after vagotomy O'Brien et al. [28]. The disruption of sympathetic efferent nerves in the intestine leads to uninhibited parasympathetic vagal efferent nerves, which in turn enhances intestinal motility Chan [29]. In this study, we used the Common Terminology Criteria for Adverse Events (CTCAE—Version 5.0) to evaluate the severity of diarrhea. No patient in either group had diarrhea of grade 3 or higher, and only 1 patient in the P-LADG group had grade 2 diarrhea, while 5 patients in the R-LADG group had grade 2 diarrhea. It has also been shown that in patients with type I diabetes and sympathetic dystrophy, the abnormally rapid passage of fluid through the distal small intestine plays an important role in the development of diarrhea [30]. Therefore, this study compared the prevalence of preoperative diabetes in the two groups and there was no significant difference between the two groups. It should be noted that in this study, although there was no significant difference in the incidence of gastritis between the two groups, the prevalence was higher in both groups, which may be related to our choice of Billroth II anastomosis. In addition, there was no significant difference in the incidence of postoperative gallstones between the two groups, possibly due to the preservation of the hepatic branch of the vagus nerve in both groups. Finally, although there was a difference in the incidence of diarrhea between the two groups, this difference was not very significant, probably due to the small sample size.

In recent years, it was found that there was no significant difference between postprandial and preprandial plasma ghrelin ratios in the vagus nerve preserved group, while it was significantly higher in the unpreserved group Takiguchi et al. [31]. Ghrelin mainly functions by stimulating appetitive signals in the hypothalamus and influencing gastrointestinal activity through vagus nerve, which may be related to delayed gastric emptying after gastric cancer surgery, for which more studies are still needed to confirm the correlation and underlying mechanisms.

This study still has many limitations. First, this study was conducted in only one medical institution with a small sample selection, and the specific role of preserving the vagus nerve's celiac branch could not be fully confirmed yet. Second, the collection for data on the incidence of various symptoms could be influenced by the follow-up period and patient's subjective situations, which lacked the support of more in-depth international questionnaires. Therefore, the role of preserving the vagus nerve's celiac branch in gastric cancer surgery still needs to be confirmed in a large-scale multicenter clinical study.

## Conclusion

In conclusion, our findings support the necessity of preserving the celiac branch of the vagus nerve in laparoscopic gastric cancer surgery. We found that it may help reduce the incidence of postoperative diarrhea and further improves postoperative quality of life in patients without showing impact on short-term postoperative outcomes or postoperative nutritional status.

## Data Availability

Data and materials are available. The datasets analysed during the current study are available from the corresponding author on reasonable request.

## References

[1] Sung H, Ferlay J, Siegel RL, Laversanne M, Soerjomataram I, Jemal A (2021). Global Cancer Statistics 2020: GLOBOCAN Estimates of Incidence and Mortality Worldwide for 36 Cancers in 185 Countries. CA Cancer J Clin.

[2] Morgan E, Arnold M, Camargo MC, Gini A, Kunzmann AT, Matsuda T (2022). The current and future incidence and mortality of gastric cancer in 185 countries, 2020–40: A population-based modelling study. EClinicalMedicine.

[3] Jun JK, Choi KS, Lee HY, Suh M, Park B, Song SH (2017). Effectiveness of the Korean National Cancer Screening Program in Reducing Gastric Cancer Mortality. Gastroenterology.

[4] Choi KS, Jun JK, Suh M, Park B, Noh DK, Song SH (2015). Effect of endoscopy screening on stage at gastric cancer diagnosis: results of the National Cancer Screening Programme in Korea. Br J Cancer.

[5] Hu Y, Huang C, Sun Y, Su X, Cao H, Hu J (2016). Morbidity and Mortality of Laparoscopic Versus Open D2 Distal Gastrectomy for Advanced Gastric Cancer: A Randomized Controlled Trial. J Clin Oncol.

[6] Zeng F, Chen L, Liao M, Chen B, Long J, Wu W (2020). Laparoscopic versus open gastrectomy for gastric cancer. World J Surg Oncol.

[7] Lee SI, Choi YS, Park DJ, Kim HH, Yang HK, Kim MC (2006). Comparative study of laparoscopy-assisted distal gastrectomy and open distal gastrectomy. J Am Coll Surg.

[8] Yano H, Monden T, Kinuta M, Nakano Y, Tono T, Matsui S (2001). The usefulness of laparoscopy-assisted distal gastrectomy in comparison with that of open distal gastrectomy for early gastric cancer. Gastric Cancer.

[9] Fukunaga T, Hiki N, Kubota T, Nunobe S, Tokunaga M, Nohara K (2013). Oncologic outcomes of laparoscopy-assisted distal gastrectomy for gastric cancer. Ann Surg Oncol.

[10] Liu Y, Cui X, Zhang Y, Cao L, Hu X (2020). Efficacy of Celiac Branch Preservation in Billroth-I Reconstruction After Laparoscopy-Assisted Distal Gastrectomy. J Surg Res.

[11] Kim SM, Cho J, Kang D, Oh SJ, Kim AR, Sohn TS (2016). A Randomized Controlled Trial of Vagus Nerve-preserving Distal Gastrectomy Versus Conventional Distal Gastrectomy for Postoperative Quality of Life in Early Stage Gastric Cancer Patients. Ann Surg.

[12] Miyato H, Kitayama J, Hidemura A, Ishigami H, Kaisaki S, Nagawa H (2012). Vagus nerve preservation selectively restores visceral fat volume in patients with early gastric cancer who underwent gastrectomy. J Surg Res.

[13] Wang CJ, Kong SH, Park JH, Choi JH, Park SH, Zhu CC (2021). Preservation of hepatic branch of the vagus nerve reduces the risk of gallstone formation after gastrectomy. Gastric Cancer.

[14] Tomita R (2009). Gastric emptying function in patients 5 years after pylorus-preserving distal gastrectomy with or without preserving pyloric and hepatic branches of the vagal nerve for early gastric cancer. World J Surg.

[15] Japanese gastric cancer treatment guidelines (2014). ver.4. Gastric Cancer..

[16] Nagano H, Ohyama S, Sakamoto Y, Ohta K, Yamaguchi T, Muto T (2004). The endoscopic evaluation of gastritis, gastric remnant residue, and the incidence of secondary cancer after pylorus-preserving and transverse gastrectomies. Gastric Cancer.

[17] Yamada H, Kojima K, Inokuchi M, Kawano T, Sugihara K (2011). Efficacy of celiac branch preservation in Roux-en-y reconstruction after laparoscopy-assisted distal gastrectomy. Surgery.

[18] Tomita R, Fujisaki S, Tanjoh K (2003). Pathophysiological studies on the relationship between postgastrectomy syndrome and gastric emptying function at 5 years after pylorus-preserving distal gastrectomy for early gastric cancer. World J Surg.

[19] Freites-Martinez A, Santana N, Arias-Santiago S, Viera A (2021). Using the Common Terminology Criteria for Adverse Events (CTCAE - Version 5.0) to Evaluate the Severity of Adverse Events of Anticancer Therapies. Actas Dermosifiliogr (Engl Ed)..

[20] Miao FJ, Jänig W, Levine JD (1997). Vagal branches involved in inhibition of bradykinin-induced synovial plasma extravasation by intrathecal nicotine and noxious stimulation in the rat. J Physiol.

[21] Yunoki Y (1995). Effects of resection of celiac and pyloric branches of vagus nerve on the interdigestive motor activity of the upper digestive tract and biliary tree. J Smooth Muscle Res.

[22] Kong SH, Kim JW, Lee HJ, Kim WH, Lee KU, Yang HK (2009). The safety of the dissection of lymph node stations 5 and 6 in pylorus-preserving gastrectomy. Ann Surg Oncol.

[23] Furukawa H, Ohashi M, Honda M, Kumagai K, Nunobe S, Sano T (2018). Preservation of the celiac branch of the vagal nerve for pylorus-preserving gastrectomy: is it meaningful?. Gastric Cancer.

[24] Kojima K, Yamada H, Inokuchi M, Kawano T, Sugihara K (2008). Functional evaluation after vagus-nerve-sparing laparoscopically assisted distal gastrectomy. Surg Endosc.

[25] Uyama I, Sakurai Y, Komori Y, Nakamura Y, Syoji M, Tonomura S (2005). Laparoscopic gastrectomy with preservation of the vagus nerve accompanied by lymph node dissection for early gastric carcinoma. J Am Coll Surg.

[26] Ichikura T, Tomimatsu S, Okusa Y, Mochizuki H (1999). Improved physical condition by limiting lymphadenectomy around the coeliac artery after distal gastrectomy for gastric cancer. Eur J Surg.

[27] Stoddard CJ, Johnson AG, Duthie HL (1984). The four to eight year results of the Sheffield trial of elective duodenal ulcer surgery–highly selective or truncal vagotomy?. Br J Surg.

[28] O'Brien JD, Thompson DG, McIntyre A, Burnham WR, Walker E (1988). Effect of codeine and loperamide on upper intestinal transit and absorption in normal subjects and patients with postvagotomy diarrhoea. Gut.

[29] Chan VW (1996). Chronic diarrhea: an uncommon side effect of celiac plexus block. Anesth Analg.

[30] Rosa-e-Silva L, Troncon LE, Oliveira RB, Foss MC, Braga FJ, Gallo JL (1996). Rapid distal small bowel transit associated with sympathetic denervation in type I diabetes mellitus. Gut.

[31] Takiguchi S, Hiura Y, Takahashi T, Kurokawa Y, Yamasaki M, Nakajima K (2013). Preservation of the celiac branch of the vagus nerve during laparoscopy-assisted distal gastrectomy: impact on postprandial changes in ghrelin secretion. World J Surg.

